# Effects of concurrent chloroquine and ethanol administration on the rat kidney morphology

**DOI:** 10.11604/pamj.2018.29.49.12471

**Published:** 2018-01-18

**Authors:** Abdurrahman Abdulkadir, Ejikeme Felix Mbajiorgu, Trust Nyirenda

**Affiliations:** 1 Department of Anatomy, College of Health sciences, Federal University Birnin Kebbi, Kebbi State, Nigeria and Division of Histology and Embryology, School of Anatomical Sciences, Faculty of Health Sciences, University of The Witwatersrand, Johannesburg, Gauteng, South Africa; 2 Division of Histology and Embryology, School of Anatomical Sciences, Faculty of Health Sciences, University of The Witwatersrand, Johannesburg, Gauteng, South Africa; 3 Department of Anatomy and Physiology, Faculty of Medicine, National University of Science and Technology, Bulawayo, Zimbabwe and Division of Histology and Embryology, School of Anatomical Sciences, Faculty of Health Sciences, University of the Witwatersrand, Johannesburg, Gauteng, South Africa

**Keywords:** Chloroquine, ethanol, computed tomography, stereology, relative medullary thickness

## Abstract

**Introduction:**

The use of antimalarial chloroquine in malaria-endemic regions of Africa is rampant and it is not uncommon to find individuals taken the drug concurrent with alcohol. Effects of anti-malarial drug chloroquine (Chq) and ethanol (Et) combination on kidney volume and function using rat model was investigated.

**Methods:**

32 adult male rats were randomly distributed into four groups of 8 rats each. Group C serve as control and received vehicle only, while Q is Chq treated only, E is Et treated and QE is Et and Chq treated. Chq was administered intraperitoneally at 1mg/100g body weight weekly and 6% Et in drinking water provided ad libitum. Urine volume was collected before the treatment began and after the treatment. After eight weeks, all animals were euthanized; kidneys were harvested and fixed in 10% neutral formalin. The fixed left kidneys were scanned with computed tomography and the scan slices were used to estimate 3-dimensional kidney volume on ImageJ.

**Results:**

Total kidney volume was none significantly increased in Q, E and QE treated compared to control groups (p = 0.5150). Also, microscopic analysis showed increased proximal tubule diameter (p = 0.1426) and epithelial hypertrophy (p = 0.2530) and significant urinary space shrinkage (p = 0.00001). The initial urine volume was not significantly different between the control and treated groups (p = 0.9864) however, following treatment urine volume was significantly reduced in QE rats group (p = 0.0029).

**Conclusion:**

The results suggest chloroquine and ethanol combination as potential cause of kidney injury through structural damage and function derangement.

## Introduction

Chronic drug administration can lead to cumulative drug toxicity to body organs especially liver and kidney. Chronic administration of chloroquine causes derangement of kidney function and histological abnormalities [1,2]. Furthermore, concurrent chloroquine and alcohol ingestion is not uncommon in Africa, with the current increase in alcohol intake. It was reported by Musabayane et al [3] that chronic concurrent alcohol and chloroquine administration causes significant derangement in kidneys electrolyte handling and also observed dilatation of the urinary space and extensive damages to the proximal tubule and collecting duct on light microscopy. This evidence might suggest increased chloroquine toxicity to the kidney when the drug interacts with alcohol and a thus create the need to have more informed ways of monitoring the toxicity. Chloroquine is a synthetic antimalarial drug developed as a response to the quest for a drug to combat the scourge of malaria around the 1940s [4]. It was, however, abandoned two decades later due to resistant malaria [5] which significantly reduce the drug efficacy on malaria. Other reasons of chloroquine use are its application in treatment of some inflammatory conditions such as rheumatoid arthritis and systemic lupus erythematosus [6]. Furthermore, chloroquine is currently being investigated for application in viral therapy and cancer chemotherapy [7, 8]. These recent therapeutic applications of chloroquine require a long-term administration usually in combination therapy and toxicity is inevitable. Even so, the majority of the poor people in sub-Saharan Africa still resort to using chloroquine for any febrile episode considered to be malaria attack [9]. The situation created a repeated cycle of chronic chloroquine intake for malaria treatment. Also, the resurgence of chloroquine-sensitive malaria in some African countries [10, 11] makes a comeback for chloroquine use in malaria treatment.

It is facetious to not acknowledge the increase in alcohol ingestion in Africa [12] and perhaps concurrent intake of the beverage with any prescribed or over-the-counter drug with no exception to chloroquine. Both ethanol and chloroquine are metabolised by the liver cytochrome P450 enzymes and ethanol is notorious for competing with drugs for these enzymes and thereby delaying the drug metabolism and elimination [13]. This tends to increase the drug half-life and might lead to more toxicity of the drug to the body. Methods used in assessing kidney health status include transabdominal ultrasound used to check the state of kidney function and total kidney volume for hypertrophy or shrinkage [14, 15]. In addition to ultrasound, other methods of quantifying total kidney volume are the use of contrast-enhanced computed tomography (CT) [16] and also the stereological technique [17]. Stereology involved the exhaustive sectioning of the kidney and then selecting representative sections using systematic uniform random sampling, then using Cavalieri principle and point counting to estimate the area of each section. Thus, the product of sections area and section thickness provides the volume of the sample fraction. The volume of sample fraction is multiplied by the number of sections in each fraction to get the total volume. Urine concentrating ability of the kidney is also a marker of kidney function used [18]. Relative medullary thickness (RMT) was reported to have a significant direct relationship with the maximum urine concentrating ability of animals. This relationship has been reported between animal species [19] and also in a post-nephrectomy experiment on a goat [14]. RMT is by measuring the length of the medulla from the corticomedullary junction to the tip of papilla referred to as the medullary thickness (MT) relative to the kidney size (KS) using the Sperber formula [18].

$$\text{RMT}=\frac{10*\text{medullary thickness}(\text{MT})}{{}^{a}\sqrt{\text{kidney size}(\mathrm{KS})}}$$

Kidney size is the product of the kidney length, breadth and thickness all measured in millimetre. Serum urea and creatinine are important biochemical markers of kidney function and also urea plays a significant role in mammalian urine concentrating mechanism [20]. The aim of this study is to envisage the toxic effects of chronic chloroquine and alcohol administration on the kidney using morphometry and stereology in relation to the biochemical markers of kidney function.

## Methods

**Animals and housing**: All animals experiment and procedures were approved by the Animal Ethics Screening Committee (AESC) of the University of the Witwatersrand (AESC 2015/11/54C). A total of 32 adult male Sprague Dawley rats average weight 405 ± 24g were bred and housed at the Central Animal Services of the University of the Witwatersrand, Johannesburg. The rats were individually housed in Perspex cages lined with saw dust and paper shreds with the temperature maintained at 21 ± 1^°^C and a 12 hour light cycle, cages were changed twice a week. Standard rat food was provided *ad libitum* to all the rats.

**Study design**: The rats were acclimatised to the cage and handling for five days and then randomly distributed into four groups of 8 rats each. A control group (C) and three experimental groups, the experiment include a group with chloroquine treatment only (Q), a group with ethanol treatment only (E) and a group with both chloroquine and ethanol treatments (QE). All treatments lasted for eight weeks. Chloroquine phosphate salt (Sigma Aldrich, PHR1258) was purchased commercially and was reconstituted in our laboratory with 0.9% saline into 10mg/ml solution; fresh solution was made for each week. Chloroquine was administered weekly for eight weeks based on the weekly weight as an intraperitoneal injection at the dose of 0.1ml/100g (equivalent of 1mg/100g) body weight to the treatment group Q and QE, while C and E groups received 0.1ml/100g body weight of 0.9% saline. Absolute alcohol was acquired from our general lab which was then diluted with distilled water to our desired concentration of 6%, this concentration is approximate to the concentration of ethanol in commercial beer. Ethanol was administered *ad libitum* in distilled water at a concentration of 6% ethanol in distilled water (V/V) to the experimental groups E and QE, while groups C and Q received plain distilled water *ad libitum* for consecutive eight weeks of the experiment. At the end of the eight weeks treatment, all rats terminal weight were taken and then were euthanised with an overdose of sodium pentobarbitone (Euthanaze^®^) 20mg/100g body weight intraperitoneal injection. Blood samples were collected using 5 ml syringes with 21G needles by cardiac puncture into plain blood sample tubes to collect serum. Kidneys were excised through midline abdominal incisions and were fixed in 10% neutral buffered formalin for 10-14 days until further analysis. The right kidney was separated from the left at the point of collection for consistency of analyses. Both kidneys were weighted using RADWAG sensitive balance (RADWAG Wagi Elektroniczne, Poland) prior to fixation.

**Urine collection and volume estimation**: Urine volume was collected over 24 hours before the injections and after the 8 weeks of treatment. Rats were transferred in metabolic cages individually for 24 hours and then all the urine produced was collected and then the rats were returned to the normal cages. Urine volume was determined using measuring cylinder.

**Relative medullary thickness measurements**: The right kidney was used for the RMT measurements; the first step was gross length, breadth and width measurements with a dial vernier calliper (Mauser Inox 0-150mm, 0.05mm). The second step is the medullary thickness (MT) measurement; this was done by dissecting the right kidney into two mid-sagittal halves. One of the halves was placed on a dissecting microscope 5x total magnification (Nikon SMZ1500, Japan) fitted with a digital camera (Nikon DSfi1) and capture screen (Nikon Digital sight) and digital images were acquired. On these images the cortex and medulla were clear in fixed kidney and a metric transparent ruler was introduced into the images for standardisation of the scale. The capture screen provides a text option to properly put a label to the images. The images were then imported into ImageJ image analysis software. On ImageJ scale was set using the metric ruler in the images and was standardised by comparing the length of the kidney measured with a vernier calliper to the length measured with the ImageJ software and it was 0.5mm accurate and was acceptable. The medullary thickness was then measured using the ImageJ software [21] from the corticomedullary junction to the tip of the papilla, together with the cortical thickness, and the results were exported from the ImageJ into Microsoft Excel 2010 for further analyses.

**Figure 1:**
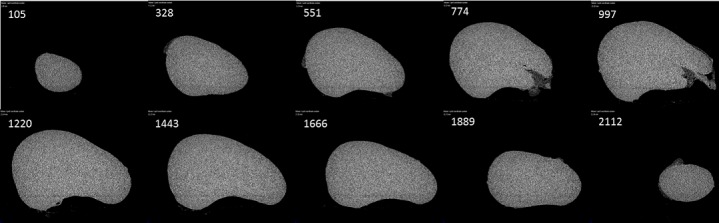
Stereological method of sampling; the figure above shows representative scan slices of the kidney used on ImageJ to estimate total volume

**Stereological volume estimation**: The 10% neutral formalin fixed left kidney was scan with a microfocus computed tomography machine (Nikon X TH 225/320 LC) at a 50KV and 100μA. The kidney was stabilised with Styrofoam above and below in a transparent container which is then exposed to the X-ray source for scanning. The scanned images were then reconstructed to a three-dimensional VGI image file with CT 3D Pro XT 2.2 software which can now be analysed with VGStudio Max 3.0 (Volume Graphics GmbH, Germany). The VGStudio provide serial sections images of the kidney which met the criteria for unbiased stereological sectioning method. Serial sections were obtained using the VGStudio software and saved as image files; the sections exhaust the whole kidney at a 10μm interval. So, depending on the kidney size thousands of sections were generated, a sample fraction was calculated and then 10 sections were sampled based on a pilot using the systematic uniform random sampling (Figure 1). Significant coefficient of error (CE) was provided by the 10 sections that were chosen. The images were analysed with Volumest plugin [22] on ImageJ [21] and the volume (mm3) and the CE were obtained for the 10 sections. Total kidney volume is the product of the volume from the 10 sections multiplied by the sample fraction. The procedure was repeated twice for each kidney and an average of the results was taken.

**Microscopic morphometry**: The left kidney was sliced into 3 equal parts and middle block was processed for routine histology. 5μm sections were cut on a rotary microtome and were stained with haematoxylin and eosin (H&E). Photomicrographs of the H&E stained sections were taken with Axioskop 2 microscope fitted with a camera Axiocam HRc2 at 63x magnification. Measurements were taken on the photomicrographs using the Zen lite analysis software. In each of the parameter for each of the treatment group and control group 60 measurements were done.

**Serum analyses**: The blood collected in the tubes was centrifuged with Rotofix 32A (Hettich Zentrifugen, Germany) centrifuging machine at 1067 x g for 10 minutes. Serum was then collected as the supernatant into the 1ml Eppendorf tubes and stored in -80°C deep freezer until further analyses. Serum urea was analysed with Reflotron analyser (Boehringer Mannheim, Germany) and the creatinine was sent to Idexx Laboratories, Johannesburg, for analysis.

**Statistical analysis**: All data were managed and graphs were constructed in Microsoft Excel 2010 and the statistical analyses were done with STATA 13.1, results are presented as Mean ± SEM. The p-value of 0.05 is considered significant. Data was tested for normality using Shapiro-wilk test, normally distributed data is analysed with one-way analysis of variance (ANOVA) while Kruskal-wallis was used for non-parametric data. Bonferroni post-hoc was used for multiple comparisons in significant One-way ANOVA and Dunn's multiple comparisons for significant Kruskal-wallis test.

## Results

**General findings**: At the end of the eight week treatment, the control group gained more weight than the treatment groups without statistical significance (One-way ANOVA, p = 0.3063). Also, the food consumption was more in the control group than in the treatment group (Kruskal-wallis, p = 0.4190). The kidney weight was however increased by 2%, 5% and 4% in Q, E and QE groups respectively compared to the control group (One-way ANOVA, p = 0.7957). While the kidney weight relative to body weight was decreased by 3% and 4% in the Q and E groups respectively compared to the control group, it was increased by 1% in the QE group compared to the control group (One-way ANOVA, p = 0.3865) (Table 1).

**Table 1:** General findings

Groups	N	TWG(g)	TFC(g)	KW(g)	RKW
**C**	8	159.25±13.04	789±128.45	3.24±0.11	0.616±0.017
**Q**	8	144.25±12.54	695±115.69	3.30±0.08	0.595±0.010
**E**	8	147.25±11.24	644±100.98	3.40±0.14	0.593±0.011
**QE**	8	126±12.82	678.22±107.28	3.36±0.13	0.621±0.015

Total Weight Gained (TWG), Total Food Consumption (TFC), Kidney Weight (KW), Relative Kidney Weight (RKW)

Abbreviations: C–Control, Q–Chloroquine treatment only, E–Ethanol treatment only, QE–and ethanol concurrent treatment

**Figure 2:**
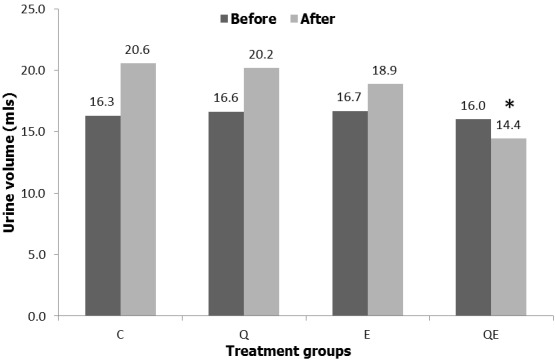
24 hour urine volume; the figure shows the changes in the urine volume collected over 24 hours in the control and experimental groups, before commencement of treatment and after the last dose of the drug

**24 hour urine volume**: Urine volume was not significantly different in both the control and treatment groups before the commencement of the experiment (One-way ANOVA, p = 0.9864), however, there was significant reduction of the urine volume (Dunns test, p = 0.0029) in QE group compared to the control group with non-significant increase in the urine volume in Q and E compared to the control group (Figure 2).

**Figure 3:**
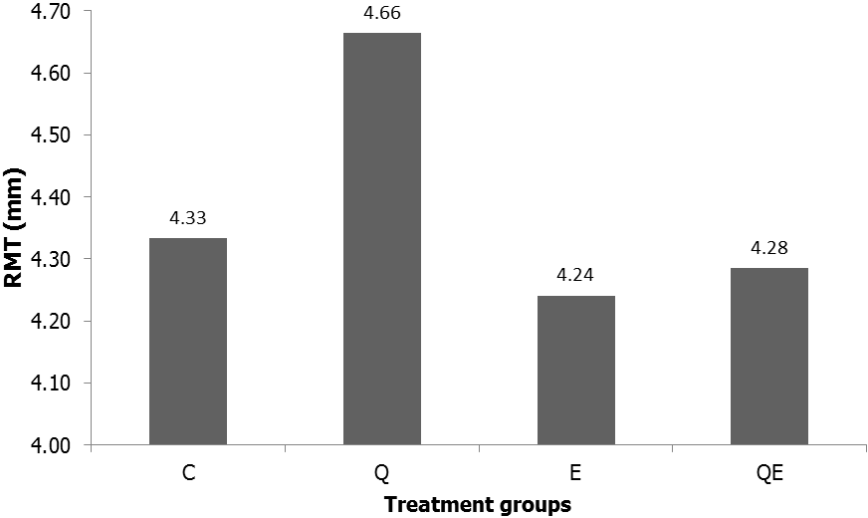
Relative medullary thickness (RMT); the figure shows the relative medullary thickness measurements in the control and treatment groups

**Relative medullary thickness**: The relative medullary thickness is increased in Q compared to control (Figure 3). It was, however, significantly decreased in E and QE compared to the control (One-way ANOVA, p = 0.0001). Cortical thickness reduced in the treatment groups compared to the control group (Table 2).

**Table 2:** The classical linear gross morphometry of the kidney

Groups	n	CT(mm)	MT(mm)	MCR
**C**	8	3.35±0.16	5.69±0.24	1.72±0.10
**Q**	8	3.30±0.10	6.25±0.29	1.91±0.14
**E**	8	3.23±0.07	5.83±0.30	1.81±0.10
**QE**	8	3.13±0.14	5.74±0.23	1.88±0.14

This table shows the linear measurements of cortical thickness (CT), medullary thickness (MT) and the medullary-cortical ratio (MCR)

Abbreviations: C–Control, Q–Chloroquine treatment only, E–Ethanol treatment only, QE–Chloroquine and ethanol concurrent treatment

**Figure 4:**
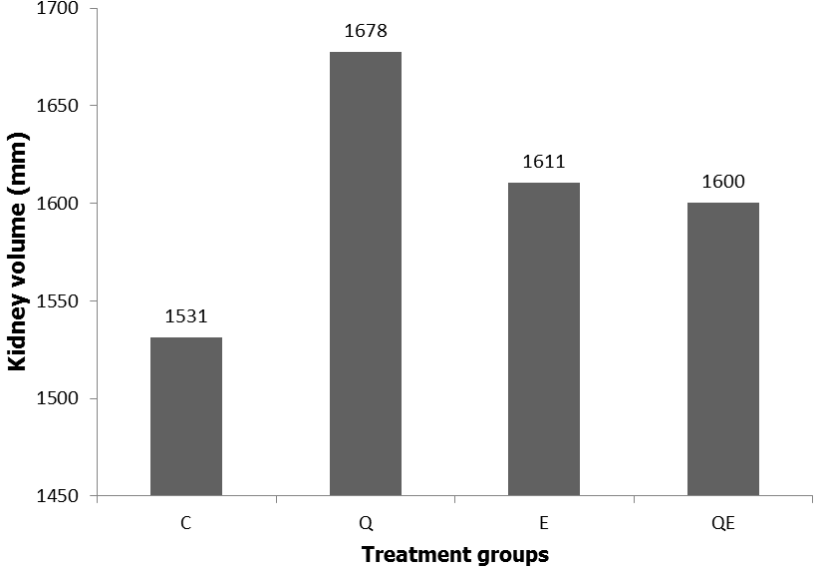
Total kidney volume; the figure shows the total kidney volume estimated using stereological technique on ImageJ volumest plugin

**Total kidney volume using stereology**: The total kidney volume was increased by 8%, 4% and 7% in Q, E and QE compared to the control group with no statistically significant differences (One-way ANOVA, p = 0.6533). The coefficient of error was 0.018 which is highly significant (Figure 4).

**Microscopic morphometry**: There was significant shrinkage of the urinary space in the treatment groups compared to the control group (One-way ANOVA, p = 0.00001), with non-significant decrease in the glomerular diameter (One-way ANOVA, p = 0.5096) and the Bowman's capsule (One-way ANOVA, p = 0.3212) (Table 3). The diameter and epithelial cells height were increased in the proximal tubules of all the treatment groups compared to the control group (One-way ANOVA, p = 0.1426, p = 0.2530 respectively for diameter and epithelial cells height) (Table 4, Table 5). The distal tubules and the collecting ducts diameters were decreased in the treatment groups compared to the control group except in the E group which showed increased diameter of the distal tubule and collecting duct (p = 0.0925, p = 0.5537 respectively) (Table 4). The epithelial cells height were surprisingly increased despite the reduced diameter of collecting ducts of the treatment groups compared to the control (One-way ANOVA, p = 0.0925), whereas there was decreased epithelial cells height of the distal tubule in treatment groups compared to the control except in the Q group (One-way ANOVA, p = 0.5537) (Table 5).

**Table 3:** Bowman's capsule and glomeruli measurements

Groups	n	GD(mm)	BCD(mm)	USD(mm)
**C**	5	0.090±0.004	0.101±0.004	0.0079±0.0004
**Q**	5	0.085±0.002	0.094±0.002	0.0048±0.0002*
**E**	5	0.089±0.003	0.100±0.003	0.0058±0.0003*
**QE**	5	0.086±0.002	0.095±0.002	0.0062±0.0003*

This table shows the measurements of the glomeruli diameter (GD), Bowman’s capsule diameter (BCD) and the urinary space diameter (USD) of the control and the treatment groups.

* significance *p*=<0.01

Abbreviations: C–Control, Q–Chloroquine treatment only, E–Ethanol treatment only, QE–Chloroquine and ethanol concurrent treatment

**Table 4:** Tubules diameter measurements

Groups	n	CDD(mm)	DTD(mm)	PTD(mm)
**C**	5	0.030±0.0012	0.037±0.0009	0.040±0.0006
**Q**	5	0.027±0.0010	0.037±0.0007	0.042±0.0008
**E**	5	0.031±0.0012	0.038±0.0008	0.042±0.0006
**QE**	5	0.028±0.0008	0.036±0.0007	0.042±0.0007

This table shows the measurements of the collecting ducts diameter (CDD), distal tubules diameter (DTD) and the proximal tubules diameter (PTD) of the control and the treatment groups

Abbreviations: C–Control, Q–Chloroquine treatment only, E–Ethanol treatment only, QE–Chloroquine and ethanol concurrent treatment

**Table 5:** Tubular epithelial cells height

Groups	n	CDE(mm)	DTE(mm)	PTE(mm)
**C**	5	0.00608±0.0003	0.00913±0.0003	0.00712±0.0002
**Q**	5	0.00617±0.0002	0.00959±0.0003	0.00773±0.0002
**E**	5	0.00618±0.0002	0.00895±0.0003	0.00747±0.0002
**QE**	5	0.00612±0.0002	0.00912±0.0002	0.00738±0.0002

This table shows the measurements of the collecting ducts epithelial cells height (CDE), distal tubules epithelial cells height (DTE) and the proximal tubules epithelial cells height (PTE) of the control and the treatment groups

Abbreviations: C–Control, Q–Chloroquine treatment only, E–Ethanol treatment only, QE–Chloroquine and ethanol concurrent treatment

**Figure 5:**
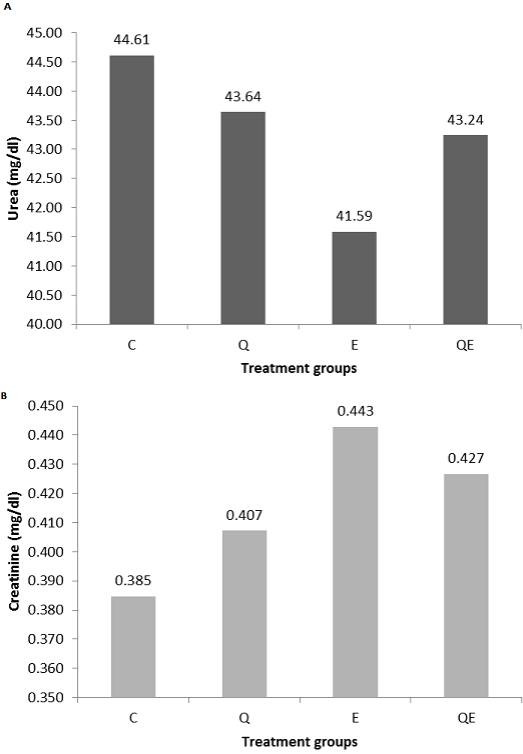
Biochemical analyses of urea and creatinine; the figure shows results of serum urea (A) and creatinine (B)

**Biochemical analyses**: The serum urea level was slightly increased in Q compared to control group, however, it was decreased in E and QE (One-way ANOVA, p = 0.9734) (Figure 5, A). Creatinine was increased in all the treatment groups compared to the control (One-way ANOVA, p = 0.1948) (Figure 5, B).

## Discussion

The results of this study suggested that chloroquine and ethanol concurrent administration causes loss of appetite evidenced by the decrease in food consumption and reduced weight gain in the treatment groups but not in the control rats. There was also increased kidney weight which is not unrelated to the increase in total volume of the organ observed in the treatment groups but not in the control rats. The increase organ weight might be due to the inflammatory response to the organ exposure to chloroquine and ethanol treatments, because chloroquine causes glomerular and tubular cell vacuolation [23], while ethanol promotes interstitial oedema and renal hypertrophy [24]. We observed renal hypertrophy in the Q, E and QE groups compared to the control group, this we believe to be the result of the proximal tubular cells hypertrophy. Alcohol is known to cause renal hypertrophy. Also, chloroquine and ethanol compromises the urine concentrating ability of the kidney as seen by significant reduction of the RMT in the treated group compared to the control. However, there was a non-significant increase in the Q group which indicates an increase in urine concentrating mechanism with chloroquine alone treatment. Ethanol was reported to cause a decrease in urine concentrating ability which conforms to our finding [25] but no such report on chloroquine alone. Some researchers [19] measure the urine osmolality in relation to RMT we, however, instead use the serum urea level which has a significant role [20] in the urine concentrating mechanism. The serum urea is seen to decrease in a similar pattern with the RMT itself, suggesting a decrease in urea level reduces the urine concentrating mechanism. Evidence from this result suggests that decrease in the urea level has a direct relationship with the RMT whereas the urine osmolality was reported to have inverse relationship. Creatinine clearance is a more clinical indicator of glomerular filtration capacity, the increase in serum creatinine level showed reduced creatinine clearance and a compromise of the glomerular filtration function. Our finding showed increased creatinine in all the treatment groups compared to the control, the increase creatinine level had an inverse relationship with the cortical thickness.

In a report by Yamashita et al [15] it was revealed that cortical thickness in a strong indicator of the renal function, as the cortical thickness tapered the function will be compromised. Significant reduction of the urine volume caused by the concurrent administration of chloroquine and ethanol is an indication of glomerular filtration compromise. Ethanol is known to increase urine volume by inhibiting the action of antidiuretic hormone (ADH) in the kidney or suppressing the blood levels of the hormone [24]. While, chloroquine is known to increase plasma level of ADH [26], the combination of chloroquine and ethanol significantly reduces urine volume suggest an intense chloroquine effect on the blood ADH levels. The general knowledge that chloroquine and ethanol both compete for the cytochrome P450 enzymes convince us that significant amount of chloroquine is maintained in the blood for a longer period when administered concurrently with ethanol. While most of the chloroquine is being metabolised by cytochrome P450 only, ethanol is metabolised by other enzymes as well [13]. With this knowledge, we believe more chloroquine will be available in the blood than alcohol, with only small amount of ethanol remaining to compete for the cytochrome enzymes. The results of concurrent chloroquine and ethanol administration showed that there is an increase in kidney weight and renal hypertrophy with compromised renal function indicated by the decrease in renal maximum urine concentrating ability and an increase in the serum creatinine level. This result provides evidence that there were actual structural changes in the kidney that altered the function and sodium water balance as reported by Musabayane et al [3]. The stereological technique used in estimation of the kidney volume is a non-bias morphometry which in itself eliminate the human bias, but also in addition to the stereology the application of microfocus computed tomography (μCT) eliminated the shrinkage factor occurring from tissue processing and also provide a more accurate sampling strategy. This method will provide more chances of using the tissue for other histological studies because after the μCT scanning the tissues were still intact.

## Conclusion

Evidence from this study suggests renal morphological changes in relation to the compromise of the function seen with concurrent administration of ethanol and chloroquine in a rat.

### What is known about this topic

- Renal fluid excretion in rats following ethanol and chloroquine administration;
- Electrolytes handling in rats following ethanol and chloroquine administration;
- Effects of ethanol and chloroquine concurrent administration on antidiuretic hormone.

### What this study adds

- Quantitative microscopic morphometry on the effects of concurrent chloroquine and ethanol on rat kidneys;
- Gross morphometry of relative medullary thickness in rat kidney;
- Application of stereology to assess kidney volume changes.

## Competing interests

The authors declare no competing interests.
